# The Platelet Response to Tissue Injury

**DOI:** 10.3389/fmed.2018.00317

**Published:** 2018-11-13

**Authors:** Felix Eisinger, Johannes Patzelt, Harald F. Langer

**Affiliations:** ^1^Section for Cardioimmunology, Department of Cardiovascular Medicine, University of Tuebingen, Tübingen, Germany; ^2^University Clinic for Cardiovascular Medicine, University of Tuebingen, Tübingen, Germany

**Keywords:** platelets, innate immunity, inflammation, infection, cytokines, leukocytes, complement, tissue remodeling

## Abstract

In recent years, various studies have increasingly explained platelet functions not only in their central role as a regulator in cellular hemostasis and coagulation. In fact, there is growing evidence that under specific conditions, platelets act as a mediator between the vascular system, hemostasis, and the immune system. Therefore, they are essential in many processes involved in tissue remodeling and tissue reorganization after injury or inflammatory responses. These processes include the promotion of inflammatory processes, the contribution to innate and adaptive immune responses during bacterial and viral infections, the modulation of angiogenesis, and the regulation of cell apoptosis in steady-state tissue homeostasis or after tissue breakdown. All in all platelets may contribute to the control of tissue homeostasis much more than generally assumed. This review summarizes the current knowledge of platelets as part of the tissue remodeling network and seeks to provide possible translational implications for clinical therapy.

## Introduction

The role of platelets in vaso-occlusive diseases such as stroke, myocardial infarction and deep vein thrombosis has long been known (1, 2). Recently, more emphasis has been placed on their influence on inflammatory and immunological processes going beyond the initiation of primary hemostasis (3). These emerging aspects include various pathologies, bacterial, and viral infections as well as cancer metastasis and many others (4). In fact, platelets interact with a broad range of immune cells and thereby help regulating the immune response to injury, infections, inflammatory responses as well as regenerative mechanisms of tissue remodeling (5, 6). Platelets are among the first cells arriving at the site of vascular lesions and organ breaches, where they interact with leukocytes, endothelial cells, and resident or circulating cells, which contribute to tissue re-organization (7). For instance, they can influence central processes such as inflammation, angiogenesis, and tissue apoptosis (8). Here, we summarize these often underestimated platelet functions and discuss them as potential targets in translational therapy in addition to the classical function of platelets as thrombus forming cells.

## Introduction to the intersection of platelet activation and tissue injury/repair

The induction and regulation of hemostasis belongs to the primary functions of platelets and can be found among the first steps of tissue repair. However, platelet activation also affects other parts of the wound healing process. For instance, tissue injury leads to the release of signaling molecules that trigger the recruitment and activation of inflammatory cells. The following immune reaction shields the organism from invading pathogens, regulates the removal of cell fragments, and damaged tissue and enables tissue restructuring. Platelets also influence this inflammatory process through a broad range of membrane receptors and soluble mediators, which are released upon platelet activation. The breakdown of tissue barriers during injury also enables the invasion of microorganisms and might provoke tissue infections. Thus, the initiation and regulation of immune reaction against invading pathogens represents an important step in tissue healing. Platelets have been shown to contribute to this process. Indeed, there is growing evidence that platelets link innate and adaptive immunity in response to infections. Angiogenesis–the formation of new vessels–is another important process in response to injury and influences tissue remodeling during wound healing, inflammation, and tumor progression. After tissue injury, new capillaries are required to provide the wound with nutrients, oxygen, and immune cells and to remove metabolites. Similar to the immune response, angiogenesis is regulated by a well-orchestrated system of cell-cell-interactions and soluble mediators. It is not surprising that platelets bearing a wide variety of membrane receptors and cytokines, also take part in the mediation of angiogenesis. Efficient recovery from tissue injury is a long process of tissue reorganization and restructuring, which is known as tissue remodeling. A growing body of evidence shows that platelets influence this elementary step of wound healing, thereby giving a possible explanation for the beneficial effects of platelets and platelet rich plasma in clinical wound therapy. In the following sections, we will give more detailed information on the different aspects of platelet response to tissue injury.

## Platelets as mediators of provisional wound closure after injury

The role of platelets in primary hemostasis is an often reviewed topic. With an average count of 150–450 × 10^9^ per liter, platelets together with erythrocytes are the most frequent cells in the blood circulation (9). Any endothelial lesion causes the exposure of collagen and tissue factor from the subendothelial matrix. Within seconds, platelets adhere to this endothelial lesion via interaction of platelet glycoprotein Ib-IX-V-receptor (GPIb-IX-V) with collagen-bound von Willebrand factor (vWF) (10). Especially at high shear rates, this first rather loose contact seems to be essential for slowing down the platelets and for enabling the formation of more stable binding by the platelet receptors α2β1 and GPVI (11). The latter adheres tightly to collagen and promotes platelet activation via an FcRγ-chain mediated mechanism (12, 13). This process leads to a rise in cytosolic calcium levels and, consequently, to the transition of platelet GPIIb/IIIa receptor from a low affinity into a high affinity state (“inside-out signaling”) (14). In addition, the platelet granule cargo containing a wide variety of highly active mediators is released, which further amplifies platelet activation and aggregation (15). Among these mediators are vWF, fibrinogen, P-selectin from α-granules, and adenosine diphosphate (ADP), calcium, and serotonin from dense granules (16–18). Furthermore, the rise in cytosolic calcium levels initiates a change of platelet shape, resulting in the formation of pseudopodia, which alters the platelet surface area and its contact with the surrounding micro-milieu (19). Finally, platelet GPIIb/IIIa receptor binds to endothelial vitronectin, vWF and soluble fibrinogen which cross-links the platelet to endothelial cells and other platelets (20, 21). In a process referred to as “outside-in signaling,” the binding of the platelet GPIIb/IIIa integrin triggers the reorganization of the cytoskeletal system via interaction with cytoskeletal proteins such as talin and kindlin-3 (22, 23). This is essential for sufficient platelet spreading, stable thrombus formation, and clot retraction (24, 25). Following activation, the platelet cyclo-oxygenase catalyzes the formation of thromboxane A2 from arachidonic acid (26). Furthermore, in the context of an endothelial lesion, platelets can be activated by a wide range of factors besides subendothelial collagen. Thrombin, produced by the simultaneously triggered coagulation cascade, is a strong activator of platelets via the platelet protease activated receptors (PAR) 1 and 4 and GPIbα (27). Additionally, ADP which is released from activated platelets and damaged tissue, further stimulates platelet activation, and aggregation by binding to the platelet purinergic receptors P2Y1, P2Y12, and P2X1 (19, 20, 28). When a patient receives antiplatelet therapy, the commonly used drugs clopidogrel, ticagrelor and prasugrel inhibit this mechanism (29–32). Another important fact is that activated platelets express P-selectin on their surface (33). This leads to the recruitment of leukocytes into the growing thrombus which as a consequence, promotes tissue factor, and fibrin deposition (34, 35). Interestingly, a recent study indicated that leukocytes might directly enhance thrombosis by the binding of leukocyte Macrophage-1 antigen (Mac-1) to the platelet GPIbα-receptor (36).

## Platelets and inflammation

**Figure 1:**
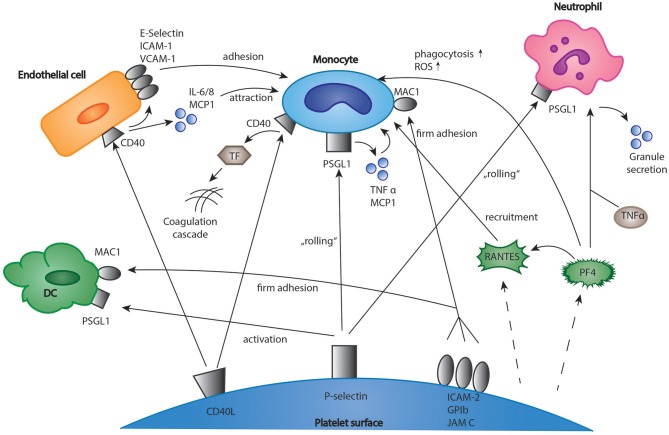
Interactions of platelets and immune cells in the regulation of inflammation. Platelets express membrane receptors and produce soluble mediators such as chemokines which regulate the inflammatory response of immune cells. The interactions include monocytes, T-cells, neutrophils, and dendritic cells. DC, dendritic cells; EC, endothelial cells; ICAM-1, intercellular adhesion molecule 1; IL 6/8, interleukin 6/8; MCP1, monocyte chemotactic protein 1; TF, tissue factor; JAM C, junctional adhesion molecule C; PF4, platelet factor 4; PSGL-1, P-selectin glycoprotein ligand-1; RANTES, chemokine ligand 5 (CCL5); TNF α, tumor necrosis factor α; VCAM-1, vascular cell adhesion molecule 1.

Upon activation, platelets expose a variety of membrane receptors, and release soluble mediators that regulate inflammation and other immune responses (2). One of the most important receptors is P-selectin, a membrane protein stored in platelet alpha-granules, which binds to P-selectin glycoprotein ligand-1 (PSGL-1) on neutrophils, monocytes, and eosinophils (37–39). Both neutrophils and monocytes are recruited to vascular lesions by “rolling” on immobilized, adherent platelets via P-selectin (Figure 1) (40, 41). More stable binding is subsequently established by the interaction of leukocyte Mac-1 and platelet receptor GPIbα, junctional adhesion molecule 3 (JAM3), and intercellular adhesion molecule 2 (ICAM-2) (42–44). This promotes recruitment of leukocytes into the growing thrombus and seems to be essential for stable thrombus formation (40, 45). Interestingly, the blockade of P-selectin significantly protects against atherosclerotic plaque formation in mice (46, 47). In the clinical Phase II trial SELECT-ACS, the P-selectin antagonist inclacumab reduced myocardial damage after NSTEMI (48). Furthermore, platelets enhance inflammation in severe asthma via P-selectin mediated recruitment of eosinophils to the lung (49). Another important mediator of the immune response is platelet CD40 ligand (CD40L), which is released both in a membrane-bound and a soluble form (50, 51). Its receptor, CD40 can be found on endothelial cells, monocytes, lymphocytes, and dendritic cells (DCs) (52). In monocytes, the binding of platelet CD40L stimulates the expression of tissue factor which activates the coagulation cascade (Figure 1) (53, 54). Furthermore, in endothelial cells, platelet CD40L upregulates the expression of the adhesion receptors E-Selectin, vascular cell adhesion molecule 1 (VCAM-1), and ICAM-1 (52) as well as the secretion of proinflammatory cytokines such as interleukin 6 (IL-6), IL-8 and monocyte chemoattractant protein-1 (MCP-1) (50, 55). Platelet released soluble CD40L seems to play an essential role for stable thrombus formation via interaction with platelet GPIIb/IIIa receptor (56). However, deficiency of platelet CD40L led to a significant reduction of atherosclerotic plaque formation in ApoE-/- mice, which could be explained by a CD40L-dependent dysregulation of T cell hemostasis (57). Apart from the membrane bound mediators, platelets secrete a broad range of cytokines (Figure 1). It was shown that upon activation, platelets synthesize and secrete IL-1β, a highly potent pro-inflammatory cytokine (58, 59). IL-1β upregulates both expression of adhesion receptors and secretion of IL-6 and IL-8 in endothelial cells and increases nitric oxide (NO) induced vascular permeability (60–62). However, other studies suggest that IL-1β secretion in platelet extracts results from contaminating leukocytes (63). Another platelet chemokine is Regulated And Normal T cell Expressed and Secreted (RANTES), which is usually released by cytotoxic T cells (64). It was demonstrated that platelet derived RANTES binds to endothelial cells and promotes the adhesion of monocytes to inflamed endothelium and atherosclerotic plaques (Figure 1) (65, 66). Furthermore, the same group demonstrated that blockade of RANTES led to reduced neointimal formation after vascular lesion in ApoE-/- mice, thereby further indicating a role of platelet-derived RANTES in the development of atherosclerosis (67). Interestingly, platelet RANTES was shown to form heteromers with neutrophil-derived human neutrophil peptide 1 (HNP-1) and platelet factor 4 (PF4), which both enhance monocyte attraction (68, 69). Recently, Machlus et al. showed that platelet RANTES induced proplatelet production in megakaryocytes—this might be an explanation for the transient rise in platelet counts during inflammation and infection (70). When referring to platelet chemokines, PF4 has to be mentioned. In 1981, Deuel et al. discovered that PF4 served as a chemoattractant both for neutrophils and monocytes (71). In following studies, however, effects of PF4 on leukocyte chemotaxis could not be underscored (72, 73). In neutrophils, PF4 enhances granule secretion in the presence of tumor necrosis factor α (TNFα) (72). Other functions of PF4 include inhibition of apoptosis in monocytes and stimulation of monocytes differentiation into macrophages (74). In addition, it stimulates monocyte oxidative burst and phagocytosis (75). As mentioned above, platelet PF4 and RANTES heteromeric interactions lead to an increased binding of monocytes to endothelial cells. Interestingly, it was demonstrated that a blockade of this interaction by the peptide inhibitor MKEY led to reduced atherosclerotic plaque formation in hyperlipidemic mice without targeting any immunological response to infections (76). A recent study indicates that MKEY also significantly reduces infarct size in strokes and improves neurological outcome (77).

## Platelets and infections

**Figure 2:**
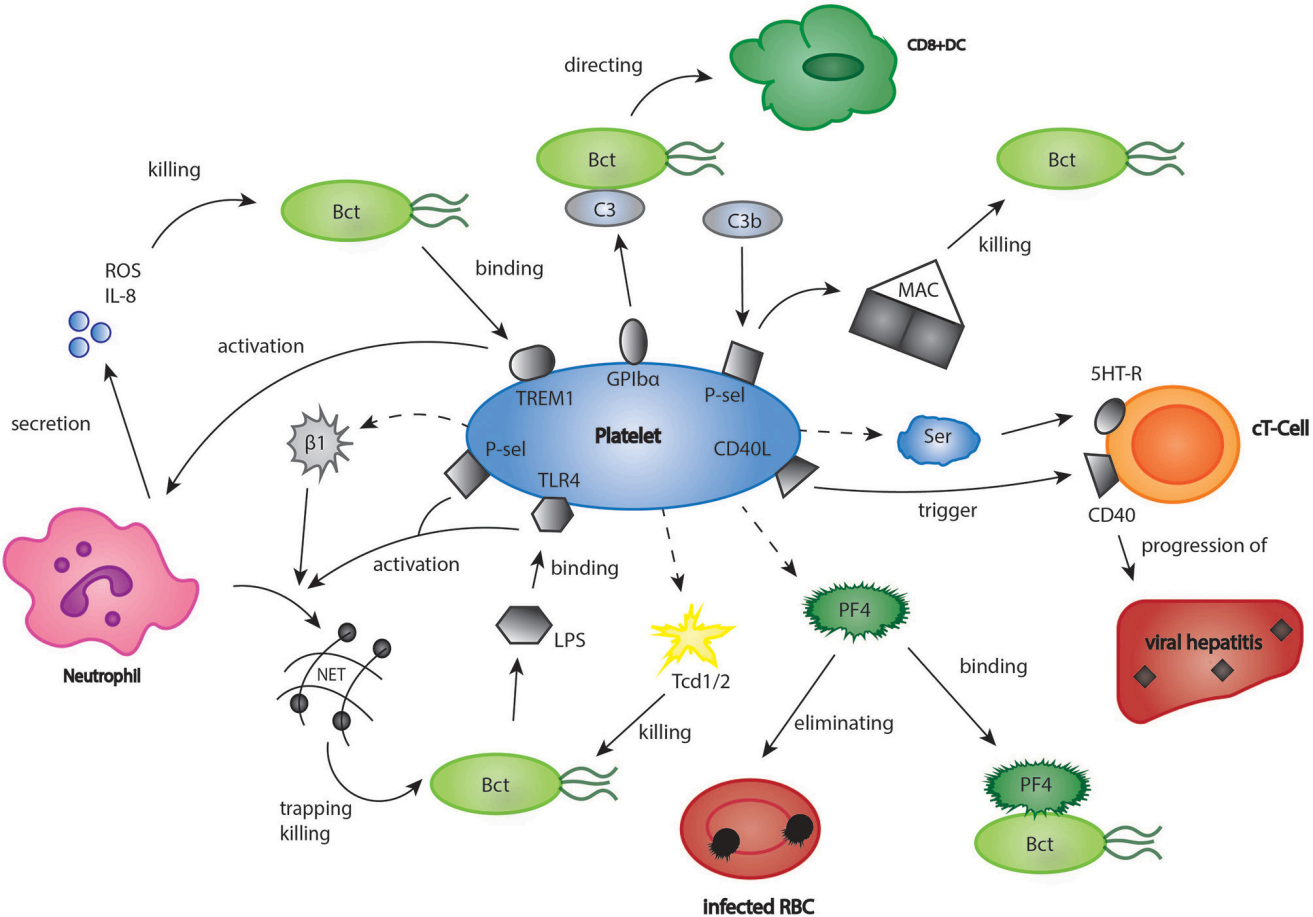
Platelets in bacterial and viral infections. The role of platelets in infections may be underestimated. Platelets were shown to have a significant part in infectious diseases such as viral hepatitis, malaria, and listeriosis. Bct, bacteria; CD40L, cluster of differentiation 40 ligand; C3, complement component 3; cT-Cell, cytotoxic T-cell; DC, dendritic cell; GPIbα, glycoprotein Ibα; 5HT-R, 5-hydroxytryptamine-receptor; IL8, interleukin 8; LPS, lipopoly-saccharides; MAC, membrane attack complex; PF4, platelet factor 4; P-sel, P-selectin; RBC, red blood cell; ROS, reactive oxygen species; Ser, serotonin; Tcd 1/2, thrombocidin 1/2; TLR4, toll like receptor 4; TREM1, triggering receptor expressed on myeloid cells 1.

There is a growing body of evidence that platelets not only influence the process of sterile thrombo-inflammation in vascular lesions, but also link innate and adaptive immunity in response to infections (7). The platelet arsenal ranges from direct killing of bacteria to enhancement of immune cell differentiation (6, 78). For instance, platelets closely interact with neutrophils, which is especially important for the formation of neutrophil extracellular traps (NET). NETs consist of DNA, histones and neutrophilic proteins and effectively trap and eliminate bacteria and fungi (79, 80). Clark et al. found that platelets recognize bacterial lipopolysaccharides (LPS) via a toll like receptor 4 (TLR 4) mediated mechanism, and as a result, stimulated NET production in neutrophils (Figure 2) (81). In a further study, it could be established that platelets contain human β1-defensins, which can trigger NET formation by neutrophils and inhibit bacterial growth (82). Platelet P-selectin was furthermore shown to play a significant role in platelet-dependent NET formation (83). Moreover, platelets express a ligand for leukocyte Triggering Receptor Expressed On Myeloid Cells 1 (TREM-1), a receptor which is upregulated in the presence of bacterial structures, and enhance TREM-1-induced respiratory burst and IL-8 secretion in neutrophils (84). T-lymphocytes represent a central cell type involved in the modulation of immune responses by platelets. In a model of acute viral hepatitis, platelets were shown to trigger cytotoxic T-cell response, thereby contributing to liver injury (Figure 2) (85) Elzey et al. demonstrated that the interaction of platelets and cytotoxic T-cells was dependent on platelet CD154 (86, 87). A further study using a model of chronic viral hepatitis showed that platelet derived serotonin significantly aggravated liver cell damage by reducing sinusoidal blood flow and impairing cytotoxic T-cell recruitment (88). Interestingly, serotonin can also activate T-cells via their 5-HT-receptors (89). Moreover, RANTES, which is secreted by platelets, seems to play an essential role in cytotoxic T-cell function during viral infections (90). In the last years, many studies have pointed out that PF4 (please refer also to the chapter platelets and inflammation) has a considerable effect in distinct models of infection (91). For instance, platelet released PF4 was proven to bind red blood cells infected with the malaria parasite *Plasmodium falciparum* and helps to eliminate the intracellular parasite (92, 93). Indeed, it was found that infected red blood cells were able to take up platelet PF4 through the Duffy antigen receptor for chemokines (DARC). Inside the erythrocyte, PF4 led to the destruction of the parasite digestive vacuolar membrane, thereby enabling its rapid elimination (94). However, some scientists have challenged these findings, as they were neither able to reproduce the inhibition of parasite growth by platelets *in vitro*, nor did they find an effect of platelet depletion on parasite blood levels (95). In contrast, another recent study examining the blood of naturally infected malaria patients demonstrated an intraerythrocytic accumulation of PF4 leading to parasite elimination as well as a platelet-dependent reduction of parasite growth *in vitro*. According to the study, between 5 and 20% of parasites in the bloodstream of malaria patients are killed by platelets (96). The contradicting results might be explained by the use of different malaria strains and parasite concentrations, which could be clarified by further, more standardized studies. Apart from its role in malaria, PF4 binds to a variety of bacterial strains thereby exposing them as a target of anti-PF4-antibodies, which leads to an enhanced phagocytosis by immune cells (Figure 2) (97). Recently, it was shown that binding of PF4 and anti-PF4-antibodies alone may kill bacterial cells (98). Interestingly, in a model of cardiac transplantation, PF4 regulated the expansion of T-cell subtype Th17, which indicated another role of platelet PF4 in the modulation of adaptive immunity (99). However, platelets can also exert a direct microbicidal effect on invading pathogens (Figure 2). In their granules, they store the proteins thrombocidin 1 and 2, which are both able to kill a broad range of bacteria (100, 101). A recently discovered link between platelets and the immune system consists of the interaction of platelets with the complement system (102–104). Thus, platelets were shown to bind C3b, one of the most central elements in the complement system, via P-selectin and to trigger the formation of anaphylatoxin C5a and the membrane attack complex (MAC), which is essential for lysis of pathogen cells (105). In addition, Verschoor et al. found that platelets adhered to bacteria opsonized by C3 via platelet GPIbα-receptor and directed them to CD8α^+^ dendritic cells in the spleen (34). Interestingly, platelet depletion led to a significantly aggravated bacterial load and reduced survival time in a model of systemic S. aureus infection (106).

However, a recent study indicated that platelets were also able to trigger an overshooting reaction to pathogens which led to a cascade of systemic shock and thrombocytopenia (107). Platelet Fcγ receptor IIA (FcγRIIA) has been identified as the major mediator of this process. Previous studies have already demonstrated that influenza virus H1N1 and several gram-positive bacteria such as Staphylococcus aureus or Streptococcus pneumoniae formed immune complexes with IgG antibodies, which bound to platelet FcγRIIA and thereby induced platelet activation (108, 109). Recent findings showed that the interaction of immune complexes with FcγRIIA stimulated platelet release of serotonin, a shock mediator initiating vasodilatation, vessel leakage and finally a systemic shock reaction. Furthermore, FcγRIIA activation led to sequestration of platelets in the lungs and the brain, which could explain transient thrombocytopenia in immune-complex induced systemic inflammation (107). Thus, platelets might be a possible target in the prevention and treatment of immune-complex triggered shock reactions.

In conclusion, these insights obtained in recent years shed more light onto the role of platelets in infections and inflammation and delineate a more complete picture of the various platelet functions beyond thrombosis.

## Platelets and angiogenesis

**Figure 3:**
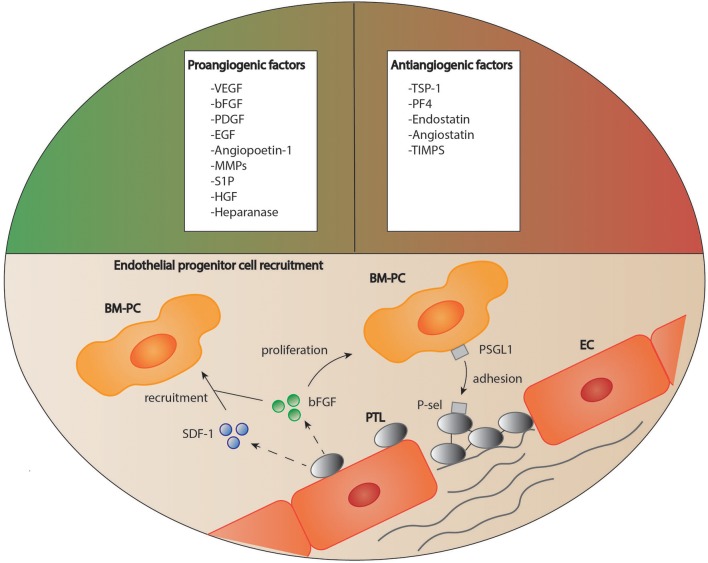
Platelets as regulators of angiogenesis. Platelets are able to both stimulate and inhibit the process of angiogenesis due to a variety of mediators stored in their granules. Furthermore, platelets are suggested to recruit circulating regenerative cells to the site of vascular lesion supported by soluble mediators. How these cells then contribute to tissue remodeling is still a matter of debate. BM-PC, bone marrow derived progenitor cells; bFGF, basic fibroblast growth factor, EC, endothelial cell; EGF, epidermal growth factor; HGF, hepatocyte growth factor; MMPs, matrix metalloproteinases; PDGF, platelet derived growth factor; PF4, platelet factor 4; P-sel, P-selectin; PSGL1, P-selectin glycoprotein ligand-1; PTL, platelet; S1P, sphingosine-1-phosphate; SDF-1, stromal cell-derived factor 1; TIMPs, tissue inhibitors of matrix metalloproteinases; TSP-1, thrombospondin 1; VEGF, vascular endothelial growth factor.

Many studies have indicated that platelets play an important role in the induction and regulation of angiogenesis after tissue injury (110). Indeed, platelet granules contain both pro- and antiangiogenic factors (Figure 3) (110, 111). It has been suggested that these angiogenic factors are sorted into different subpopulations of platelet α-granules according to their function, and that these distinct factors can be released in an agonist-dependent manner (112, 113). For instance, Italiano et al. indicated that ADP stimulated platelets secreted the proangiogenic vascular endothelial growth factor (VEGF) while thromboxane stimulation led to the release of antiangiogenic endostatin (114). In other studies, neither a functional packaging of angiogenic factors nor a selective release could be detected (115, 116).

Platelets contain a variety of factors which are essential for the stimulation of angiogenesis, such as VEGF, basic fibroblast growth factor (bFGF), epidermal growth factor (EGF), sphingosine-1-phosphate (S1P), angiopoietin-1 (Ang1), and platelet-derived endothelial growth factor (PD-ECGF; Figure 3) (117–122).

In the early stage of angiogenesis, during vessel sprouting, VEGF is the most important among them. Indeed, endothelial cells are activated by the binding of VEGF to their VEGF receptor 2 (VEGFR 2) and develop into “tip cells,” motile cells exposing filopodia that promote vessel sprouting along a VEGF gradient (123). The tip cells are followed by endothelial stalk cells, which proliferate and establish a stable vessel lumen (124). Platelets are a source of VEGF for this process. It has been shown previously that platelets stored VEGF and released it upon thrombin activation. In support of these data, Arisato et al. found significant levels of VEGF in the fibrin net after thrombus formation (117). Another recent study suggested that platelet VEGF release and concomitant angiogenesis promotion could be suppressed by treatment with Tamoxifen, an estrogen receptor modulator used for breast cancer therapy (125).

Apart from VEGF, bFGF is another essential factor for the induction of angiogenesis which can be found in platelets. When co-cultured with platelets *in vitro*, endothelial cells showed both prolonged survival and enhanced proliferation. This influence has been reduced by antibody blockade of bFGF alone, while double inhibition of bFGF, and VEGF almost abrogated it (118). Some studies indicated that bFGF exerted its proangiogenic effect through stimulation of VEGF release from endothelial cells (126). Most recent antiangiogenic therapies therefore intend to synergistically block both factors (127).

During early angiogenesis, chemoattractant factors are key elements for the formation and guiding of new vessels. Some of them can be also found in platelets, for instance S1P and PD-ECGF. Upon activation, platelets release S1P from their granules, which triggered a strong chemotactic response in endothelial cells *in vitro* (120, 128). However, in an *in vivo* cornea model, only the combination of S1P with bFGF, but not S1P injection alone showed a significant proangiogenic effect. Therefore, S1P rather appears to have a complementary role in angiogenesis stimulation. Another platelet-released chemoattractant factor is thymidine phosphorylase (TP), an enzyme first isolated from human amniochorion, which is identical with PD-ECGF (122, 129, 130). PD-ECGF exerted chemotactic effects on endothelial cells *in vitro* and promoted angiogenesis *in vivo* (131, 132). The angiogenic effects of PD-ECGF could be explained by the enzymatic release of the endothelial-cell chemoattractant 2-deoxy-d-ribose (132, 133). In addition, high levels of PD-ECGF were also associated with an increased secretion of angiogenic factors such as IL-8 and bFGF (134). Catalytic production of deoxyribose-1-phosphate (dRP) from thymidine has been identified as another mechanism of PD-ECGF-dependent induction of angiogenesis. dRP led to an upregulation of integrin α_v_β_3_ in endothelial cells and thereby stimulated cell migration *in vitro* as well as vessel formation *in vivo* (135). Apart from platelets, enhanced expression of PD-ECGF can also be found in solid tumors, such as breast carcinoma, non-small-cell lung cancer, renal cell carcinoma, and uterine cervical cancers and has been associated with increased tumor growth and higher tumor vascularization (136–139). A recent study shed light to a yet unknown function of PD-ECGF, namely that it could also activate platelets and contributed to thrombosis, which made it a possible target for novel antithrombotic therapies (140).

While platelet PD-ECGF and S1P contribute to chemotactic migration of endothelial cells, matrix metalloproteases (MMP 1, 2, and 9) facilitate this migratory process through degradation of inhibiting structures, such as the basement membrane and extracellular matrix components. Platelets have been also found to stimulate MMPs release from leukocytes and to secrete several MMPs such as MMP-1, MMP-2, and MMP-14 by themselves, thereby further promoting endothelial cell migration (141, 142).

In the later stages of angiogenesis, the stabilization of the newly formed vessels becomes more important. Thrombin-activated platelets release Ang1, an angiogenic factor which enhances vascular stability and prevents vessel leakage (121). Since vascular growth initiated by VEGF often leads to instable and leaky vessels, the effects of Ang1 are needed to promote vascular maturation (143). A recent study found that Ang1 also bound and blocked thrombomodulin, a central coagulation inhibitor (144). Although no differences in tail bleeding times could be observed between wild type and Ang1 knockout mice, this might indicate a possible role for angiogenic factors in hemostasis. Beyond the effects of Ang1, the proliferation, and migration of pericytes, e.g., smooth muscle cells and fibroblasts, to the sprout is indispensable for the stabilization of any developing vessel. Platelets support pericyte recruitment by secretion of platelet-derived growth factor BB (PDGF-BB), a strong mitogen, and chemoattractant for a variety of mesenchymal cells (145, 146). However, the effects of PDGF-BB also depend on which isoform of the PDGF receptors it is binding to.While PDGF receptor β induces mesenchymal cell proliferation, ligation to PDGF receptor α has been found to inhibit the pro-angiogenic effects of bFGF both *in vitro* and *in vivo* (147). This indicates that PDGF has a dual role in the regulation of angiogenesis and that differential receptor expression determines the outcome of PDGF signaling.

Apart from their stimulatory effect on endothelial and mesenchymal cell proliferation and migration, platelets also regulate angiogenesis through the recruitment of endothelial progenitor cells and stem cells. For instance, platelets bind to CD34^+^-stem cells via expression of stromal-derived factor 1 (SDF-1) and support their differentiation into endothelial progenitor cells (Figure 3) (111). Under hypoxic conditions, platelets recruit bone marrow derived cells to the site of vascular proliferation and thereby stimulate angiogenesis (148).

Angiogenesis, as many other physiologic processes, depends on a balance between stimulatory and inhibitory signals. In the case of an overshooting stimulation, such as in tumor angiogenesis, an excessive, “unhealthy” growth of vascular structures leads to the formation of unstable, often immature blood vessels (149). Platelets store a broad range of inhibitors of angiogenesis, among them thrombospondin-1 (TSP-1), endostatin and PF4, which block the interaction of proangiogenic VEGF with endothelial cells (110, 150, 151). These angiogenesis-restricting factors might be especially important for the regulation of tumor progression. For instance, platelet-released TSP-1 has been found to suppress tumor growth in mice inoculated with lung carcinoma cells through the inhibition of tumor angiogenesis (150). Other studies suggested that platelets might be able to scavenge angiogenic factors in tumor environment. Platelets from mice bearing liposarcoma contained higher levels of angiogenesis modulators such as VEGF and bFGF than healthy animals (152). Further studies have to clarify whether this sequestration of angiogenesis factors shows an effect on tumor vessel growth.

Indeed, many efforts have been made to elucidate the role of platelets in tumor angiogenesis (153). It is well established that tumor cells activate platelets and the coagulation cascade through the production of various procoagulant factors, the most important among them being tissue factor (TF) (154). One consequence of this TF-mediated platelet activation is an increased rate of thrombotic events and thrombophlebitis in cancer patients, clinically known as Trousseau syndrome (155, 156). Upon activation, platelets release a variety of pro-angiogenic factors such as VEGF and bFGF, which contribute to tumor angiogenesis. Indeed, the exposure of platelets to breast cancer cells triggered the secretion of VEGF and promoted increased vascular growth in a capillary tube formation assay (114). It might therefore be an interesting approach to modulate platelet activation and angiogenic factor secretion in cancer treatment. There is increasing evidence in the literature on the beneficial effects of both anticoagulation and antiplatelet therapies on tumor angiogenesis. Treatments with heparin, fondaparinux or PAR1 antagonists significantly reduced platelet release of VEGF in the presence of tumor cells and almost totally diminished platelet proangiogenic activity (157). The common COX-inhibitor aspirin has also been suggested to be a potent inhibitor of platelet-induced angiogenesis. After pre-treatment with aspirin, platelet proangiogenic effect in response to thrombin has been almost completely blocked (158). If platelet-mediated angiogenesis is inhibited by antiplatelet therapy, this might also have implications for the treatment of other diseases apart from cancer, for instance cardiovascular diseases. However, in clinical therapy, it appears difficult to separate the beneficial antithrombotic effect of aspirin from its potential influence on angiogenesis, which limits therapeutic consequences.

In general, most forms of wound healing after tissue injury are affected by platelet induced angiogenesis. While the relative importance of the different angiogenic factors is yet to be clarified, the overall influence of platelets on angiogenesis seems to be beneficial for efficient tissue regeneration. In a cornea angiogenesis model, thromobocytopenic mice showed fewer vessels, and higher vessel fragility compared with the control animals. Increased vessel fragility has also been found in thrombocytopenic mice after s.c. matrigel implantation (159). Therefore, platelets or platelet microparticles (PMP), small vesicles shed from platelet plasma membrane, could have a therapeutic potential in the treatment of tissue injuries, especially in cardiovascular diseases. There is evidence that injection of PMPs into chronic ischemic myocardium promotes capillary growth and new vessel formation in the ischemic tissue (160). In line with this, another group demonstrated increased vessel density and improved left ventricular function in pigs which received an intramyocardial injection of platelet rich plasma (PRP) and anti-inflammatory factors after myocardial infarction (161). Injection of PRP might also be beneficial in other fields. In mice suffering from open abdominal wounds, administration of platelet rich plasma gel significantly improved neovascularization, and wound healing (162). Further studies have to evaluate the safety and sustainability of these promising therapeutic approaches.

Apart from the use of platelets in regenerative medicine, platelet angiogenic factor release might also be differentially targeted in the prevention of ischemic diseases. Recently, it was shown that VEGF and bFGF, but not PDGF-BB could be held accountable for the formation of immature and leaky vessels in arteriosclerotic plaques of rabbits on a high cholesterol diet (163). Since several studies have indicated that the release of VEGF from platelets was triggered by ADP-dependent platelet activation, the use of ADP receptor inhibitors might have beneficial effects on the prevention of instable arteriosclerotic plaques. Further research will be needed to clarify this topic.

## Evidence for platelets as mediators of tissue remodeling

The role of platelets in wound healing is well established. In clinical therapy, platelet rich plasma has become an essential treatment of acute wounds, non-healing ulcers and orthopedic diseases (164–166). Tissue remodeling, which means the reorganization and restructuring of tissue, is an important step of wound healing. There is growing evidence that platelets are also involved in this process. Indeed, Schleicher et al. were able to demonstrate that activated platelets expressed FAS ligand, an apoptosis inducing ligand usually present on cytotoxic T-cells, and that blocking this platelet ligand or platelet depletion resulted in reduced apoptosis in models of retinal inflammation and stroke (167). Interestingly, Langer et al. reported enhanced apoptosis of dendritic cells after coincubation with platelets (168). Another study documented increased endothelial cell apoptosis in the presence of activated platelets (169). In a sepsis model, platelets induced apoptosis in splenocytes in a contact-dependent manner, which could be blocked by inhibition of platelet GPIIb/IIIa receptor (170). However, the role of platelets in apoptosis is multidimensional, as platelet cytokines such as S1P or PDGF show strong antiapoptotic effects, for instance providing survival of fibroblasts and human embryonic stem cells (171, 172). A second, essential platelet function in tissue remodeling is the interaction with progenitor cells. Massberg et al. indicated that platelets recruited bone marrow derived progenitor cells to a vascular injury site via secretion of SDF-1 and the P-selectin/PSGL-axis (173) SDF-1 was also shown to enhance the recruitment of smooth muscle cell progenitor cells (174) and after myocardial infarction, to augment migration of cardiac stem cells to the myocardium (175, 176). As reported in a recent study, platelet derived SDF-1 promoted alveolar regeneration after lobectomy of the lung (177), while Langer et al. found that platelet bFGF supported mesenchymal stem cell recruitment and integration into an endothelial monolayer *in vitro* (178). Other data suggest that apoptotic myocardial cells induce migration of mesenchymal stem cells via release of hepatocyte growth factor (HGF) and that this process is inhibited by platelet expression of the inflammatory cytokine high mobility group box 1 (HMGB1) (179, 180). Therefore, platelet-progenitor cell interactions seem to be complex and multidimensional. Another currently discovered platelet function raises interesting questions about the role of platelets in the stabilization of vascular integrity. Two platelet receptors, GPVI and C-type lectin−2 (CLEC-2) are associated with immunoreceptor tyrosine-based activation motive (ITAM) mediated signaling (181). Binding of the respective ligands collagen or podoplanin to these receptors leads to the phosphorylation of the ITAM tyrosine residues, which triggers an intracellular signaling cascade and finally results in platelet activation (182). It was shown that both deficiencies of GPVI or CLEC-2 expression and the blockade of the intracellular ITAM signaling pathway significantly increased vascular permeability during inflammation. Thus, ITAM-mediated platelet activation seems to be essential for the maintenance of vascular integrity under inflammatory conditions (183). In line with that, platelet-depletion resulted in hemorrhage in different models of inflammation but not in non-inflammatory control groups (184). A recent study suggested that neutrophil invasion triggered bleeding in thrombocytopenic mice, whereas GPVI-mediated platelet recruitment prevented this complication (185). Platelet CLEC2 receptor also stabilizes the vascular integrity. Current data indicate that it also exerts an anti-inflammatory effect in sepsis and promotes thrombus development in hemostasis, which makes CLEC2 an interesting target for antithrombotic therapy (186, 187).

Platelets have been also shown to affect specific organ regeneration after injury. In the liver, hepatocyte proliferation was markedly reduced in thrombocytopenic mice after 70% hepatectomy, but could be completely restored by injection of serotonin receptor agonists. Concomitantly, mice lacking platelet serotonin also presented with reduced liver regeneration markers (188). In a model of ischemic/reperfusion hepatic injury, platelet depletion led to increased liver cell necrosis after 7 days while absence of platelet serotonin significantly diminished hepatocyte proliferation (189). Another beneficial effect of platelet serotonin has been demonstrated in a study of regeneration in the older liver. A serotonin agonist stimulated hepato-sinusoidal blood flow and hepatocyte proliferation through the enhanced expression of proangiogenic VEGF (190). Platelets also release S1P, which suppressed apoptosis in human liver sinusoidal endothelial cells *in vitro* and promoted as well the release of VEGF (191). In a recent study, platelets were demonstrated to activate liver sinusoidal endothelial cells via SDF-1 and to further upregulate this process through VEGF-mediated myeloid cell recruitment (192). Although this mechanism contributed to liver regeneration, platelet derived SDF-1 also played a role in the pathogenesis of liver fibrosis. In chronic injury, SDF-1 receptor expression switched from the pro-regenerative C-X-C chemokine receptor type 7 (CXCR7) to the pro-fibrotic CXCR4, thereby initiating liver fibrosis (193). Platelets also regulate organ remodeling after lung injury. Similarly to liver regeneration, platelet released SDF-1 plays a pivotal role in this process. A recent study found that after lobectomy, platelet SDF-1 bound to CXCR4 and 7 on pulmonary capillary endothelial cells, which resulted in the secretion of mediators that stimulated alveolar regeneration (177). After bleomycin-induced lung injury, SDF-1/CXCR4 axis mediated stem cell recruitment from the bone marrow to the lungs and contributed to lung fibrosis (194). Platelets are also involved in inflammatory airway remodeling in chronic asthma, namely, platelet depletion significantly decreased subepithelial fibrosis and smooth muscle thickening in the airway wall (195). This is consistent with the results of a study on platelet induced fibrosis, which indicated that platelet released serotonin stimulates extracellular matrix production in fibroblasts (196). These effects can also be found in the heart tissue after myocardial infarction. A recent study showed that high levels of platelet activation after ST-segment elevation myocardial infarction (STEMI) predicted the risk of adverse left ventricular remodeling (197). Patients with aortic stenosis showed elevated serotonin blood levels which were strongly correlated with left ventricular hypertrophy. As platelets are the major source of serotonin outside the CNS, this might suggest a causal role of platelet activation and serotonin release in myocardial remodeling. Another platelet derived mediator in cardiac fibrosis and hypertrophy is transforming growth factor beta 1(TGF-β1), which is stored, and released from α-granules upon platelet activation. After surgical transverse aortic constriction (TAC), mice specifically lacking platelet TGF-β1 showed a significant reduction of cardiac hypertrophy and fibrosis as well as better cardiac function than wild type animals (198). At the same time, mice deficient in platelet activating receptor P2Y12 showed reduced cardiac fibrosis after TAC (199). Several studies implicated a protective effect of platelets on heart function after injury. By using a TAC model, Wu et al. found that perivascular coronary fibrosis and smooth muscle proliferation was significantly enhanced after platelet depletion (200). However, this response might also be a result of a reactive rise in platelet counts following the treatment. Another study examined the effects of platelet releasates on cardiomyocte survival after ischemic injury *in vitro* and discovered a protective effect of platelet SDF-1α and TGF-β1 (201).

In summary, there is striking evidence that platelets contribute to tissue regeneration and remodeling after injury in different organs. Indeed, platelet cytokines and mediators regulate pivotal elements of tissue remodeling such as angiogenesis, apoptosis, stem cell recruitment and connective tissue production. While in angiogenesis, platelets show an overall angiogenic effect which might be useful for treatment of ischemic injury, their role in apoptosis has not been well established yet. Future studies have to clarify whether platelet induced apoptosis contributes to the healing process or on the contrary, aggravates cell damage. With respect to stem cell recruitment, several studies have outlined the beneficial effects of platelets on wound healing and vascular formation through SDF-1 and bFGF secretion (111, 202). Furthermore, platelet mediators such as SDF-1 and TGF-β1 stimulate extracellular matrix formation and connective tissue restructuring. While this represents a necessary step in tissue remodeling, it can also lead to tissue fibrosis and hypertrophy. Both in acute liver and lung injury, platelet cytokines contributed to the restoration of tissue homeostasis. However, in case of chronic injury, platelet activation provoked a pivotal stimulation of fibrosis and after ischemic heart injury, activated platelets have been associated with enhanced ventricular hypertrophy and fibrosis. Despite these controversial results, platelet rich plasma is a well-established therapy to treat chronic wounds and ulcers. The effects of the platelets on tissue remodeling depend on the affected organ which needs to be considered when developing new therapeutic targets.

## Conclusions

In conclusion, platelets are mediators between various closely related processes such as inflammation-immunity, inflammation-angiogenesis, and hemostasis-inflammation.

Consequently, platelets seem to be a central part of the network counteracting tissue breakdown and pathogen invasion. There is increasig evidence for their role in tissue remodeling ranging from the induction of apoptosis and the recruitment of progenitor cells to the maintenance of vascular integrity. A broad range of basic research, translational approaches and clinical studies are still required to improve our understanding about the underlying mechanisms as to how platelets modulate the progression of and the regeneration from diseases, in order to use this knowledge for targeted treatment.

## Author contributions

FE wrote the manuscript. JP wrote parts of and corrected the manuscript, HL conceptualized, wrote and corrected the manuscript.

### Conflict of interest statement

The authors declare that the research was conducted in the absence of any commercial or financial relationships that could be construed as a potential conflict of interest.
